# Functional Antagonism between Sas3 and Gcn5 Acetyltransferases and ISWI Chromatin Remodelers

**DOI:** 10.1371/journal.pgen.1002994

**Published:** 2012-10-04

**Authors:** Anne Lafon, Emily Petty, Lorraine Pillus

**Affiliations:** 1 Division of Biological Sciences, University of California San Diego, La Jolla, California, United States of America; 2 UCSD Moores Cancer Center, La Jolla, California, United States of America; University of California San Francisco, United States of America

## Abstract

Chromatin-modifying enzymes and ATP-dependent remodeling complexes have been intensely studied individually, yet how these activities are coordinated to ensure essential cell functions such as transcription, replication, and repair of damage is not well understood. In this study, we show that the critical loss of Sas3 and Gcn5 acetyltransferases in yeast can be functionally rescued by inactivation of ISWI remodelers. This genetic interaction depends on the ATPase activities of Isw1 and Isw2, suggesting that it involves chromatin remodeling activities driven by the enzymes. Genetic dissection of the Isw1 complexes reveals that the antagonistic effects are mediated specifically by the Isw1a complex. Loss of Sas3 and Gcn5 correlates with defective RNA polymerase II (RNAPII) occupancy at actively transcribed genes, as well as a significant loss of H3K14 acetylation. Inactivation of the Isw1a complex in the acetyltransferase mutants restores RNAPII recruitment at active genes, indicating that transcriptional regulation may be the mechanism underlying suppression. Dosage studies and further genetic dissection reveal that the Isw1b complex may act in suppression through down-regulation of Isw1a. These studies highlight the importance of balanced chromatin modifying and remodeling activities for optimal transcription and cell growth.

## Author Summary

In eukaryotes, essential processes such as transcription, replication, and repair of damage occur in the context of chromatin. The structure of chromatin is tightly regulated during the cell cycle by chromatin-modifying enzymes, including acetyltransferases, and ATP-dependent remodeling complexes. Although there has been extensive characterization of their individual functions, little is known about how their activities are coordinated to maintain cell viability. In this study, we show that the critical loss of Sas3 and Gcn5 acetyltransferases can be functionally rescued by inactivation of ISWI remodelers. At a molecular level, the effects on cell viability tightly correlate with the recruitment of RNA polymerase II (RNAPII) at active genes, suggesting that transcriptional regulation may be the mechanism underlying cell viability rescue. Our genetic analyses reveal distinct roles for the two Isw1a and Isw1b complexes; in particular, the antagonistic effects are mediated specifically by the Isw1a complex. These studies highlight the importance of balanced chromatin modifying and remodeling activities for optimal transcription and cell growth.

## Introduction

Two major classes of enzymes regulate the architecture of chromatin and are thereby critical for DNA-templated processes such as transcription, replication, and repair of damage. Remodeling enzymes use the energy of ATP hydrolysis to alter the structure or position of nucleosomes (reviewed in [1], [2]), whereas chromatin modifying enzymes act post-translationally on multiple nuclear substrates. Prominent among these are the nucleosomal histones that are extensively modified on their N- and C-terminal tails (reviewed in [3]). Covalent modifications of histones and other chromatin proteins are diverse, including at least six specific types of reversible and dynamic modifications that are catalyzed by multimeric enzyme complexes. Among the consequences resulting from histone modifications, two have been especially well characterized. The first is disruption of contacts between DNA and histones or between nucleosomes. In this case, lysine ε-acetylation can destabilize nucleosomal interactions since it neutralizes this otherwise charged residue. The second consequence involves recruitment of effector proteins that bind via conserved recognition domains. For example, histone acetylation can be recognized by bromodomains, whereas histone methylation is recognized by chromo-like-domains and PHD domains (reviewed in [4]). These domains are found in many nuclear proteins, including chromatin modifying enzymes and remodeling complexes.

The simultaneous existence of multiple different marks on histones has led to the recognition that crosstalk among modifications can be a critical determinant for regulation of gene expression [5]. In addition, cooperation between histone modifiers and ATP-dependent remodeling complexes can contribute to transcriptional regulation (reviewed in [6]–[8]) and DNA damage repair (reviewed in [9]). For example, the histone acetyltransferase (HAT) Gcn5 and the SWI/SNF chromatin remodeling complex were proposed early on to have cooperative functions in transcriptional activation by working in concert to modify chromatin structure [10]–[12]. Indeed, histone acetylation mediated by the SAGA complex containing Gcn5 stabilizes the anchoring of SWI/SNF to nucleosomes at promoters, and thus is important for SWI/SNF-dependent nucleosome remodeling and transcriptional activation *in vitro* and *in vivo* [13], [14]. The SAGA complex interacts with another chromatin remodeling factor, the chromodomain protein Chd1 [15], [16], which is a component of Gcn5-containing SAGA and SLIK/SALSA HAT complexes [17]. In addition, Gcn5 is functionally linked to the essential RSC chromatin remodeling complex: H3K14 acetylation is recognized by one essential tandem bromodomain of Rsc4 and contributes to RSC complex-dependent gene activation [18], [19].

Acetylation of histone H3 at lysines 9 and 14 strongly correlates with transcriptional activity and peaks over start sites of active genes [20]. Gcn5 is the HAT responsible for the majority of this acetylation *in vivo* [21]–[23], consistent with the observation that Gcn5 is generally recruited to promoters of active genes, as described for H3K9 and K14 acetylation marks [20], [24]. Two other HATs also specifically target histone H3 at K9 and K14 *in vivo*: the MYST family HAT Sas3 (reviewed in [25]), and Elp3 [21], [23]. Their functions appear most critical in the absence of Gcn5. In particular, although inactivation of Sas3 in otherwise wild-type cells does not elicit obvious phenotypes, diminished Sas3 activity in a *gcn5*Δ null mutant results in defects in cell cycle progression, and complete loss of activity leads to cell death [22]. Genome-wide mapping established that Sas3 and Gcn5 are recruited to many of the same actively transcribed genes [26]. Furthermore, the binding sites of these HATs correlates with the H3K14 acetylation mark [26]. These observations strongly suggest that Sas3 and Gcn5 acetyltransferases are critical for active transcription, although the molecular mechanisms underlying their regulation have not been fully elucidated. In particular, since mutations of the established target lysines in histone H3 result in only mild phenotypes [27], [28], the essential function revealed in *gcn5*Δ *sas3* mutants most likely extends beyond acetylation of H3.

Functional links have not yet been established between Sas3 and chromatin remodeling, or the loss of viability that results when both Sas3 and Gcn5 activities are compromised. We describe here a critical interaction between Sas3 and Gcn5 acetyltransferases and ISWI chromatin remodelers. Strikingly, and in contrast with nucleosome disrupting remodelers such as SWI/SNF and Chd1, inactivation of Isw1 or Isw2 relieved conditional lethality in a *gcn5*Δ *sas3* mutant. Genetic dissection of the complexes through which Isw1 acts clearly reveals that the antagonistic effects are mediated through the Isw1a complex, and furthermore, that elimination of non-catalytic subunits of Isw1b can overcome this antagonism. At a molecular level, the effects on cell viability tightly correlate with the recruitment of RNA polymerase II (RNAPII) at active genes. Together these studies provide new evidence for functional distinctions between the families of chromatin remodeling activities and point to critical interactions between the Sas3 and Gcn5 acetyltransferases, ISWI remodeling machines, RNAPII recruitment, and chromatin compaction.

## Results

### Functional antagonism between Sas3 and Gcn5 acetyltransferases and ISWI chromatin remodeling enzymes

Among the major H3 acetyltransferases, either Gcn5 or Sas3 is required for cell viability: loss of both enzymes leads to death. To understand this loss of viability, we asked if other chromatin-modulating activities contributed to it, and in particular if there was a role for ATP-dependent chromatin remodeling activities.

There are four distinct families of biochemically defined chromatin remodeling complexes: SWI-SNF, ISWI, CHD, and INO80 [1]. The RSC and INO80 catalytic ATPases Sth1 and Ino80 are essential, although the *ino80Δ* lethality appears restricted to the W303 genetic background [29], [30]. Individual inactivation of the other catalytic ATPases, Snf2, Isw1, Isw2 or Chd1 does not trigger marked growth defects in otherwise wild-type cells [15], [31], [32]. However, earlier studies reported synthetic lethality between SWI-SNF components and members of the Gcn5-SAGA complex [11].

We began by evaluating the effects of ATP-dependent chromatin remodeling activities when Sas3 and Gcn5 activities were compromised, using the temperature-sensitive *gcn5*Δ *sas3* conditional mutant described earlier [22]. We observed two types of functional interactions between chromatin remodelers and the Sas3 and Gcn5 acetyltransferases.

**Figure 1:**
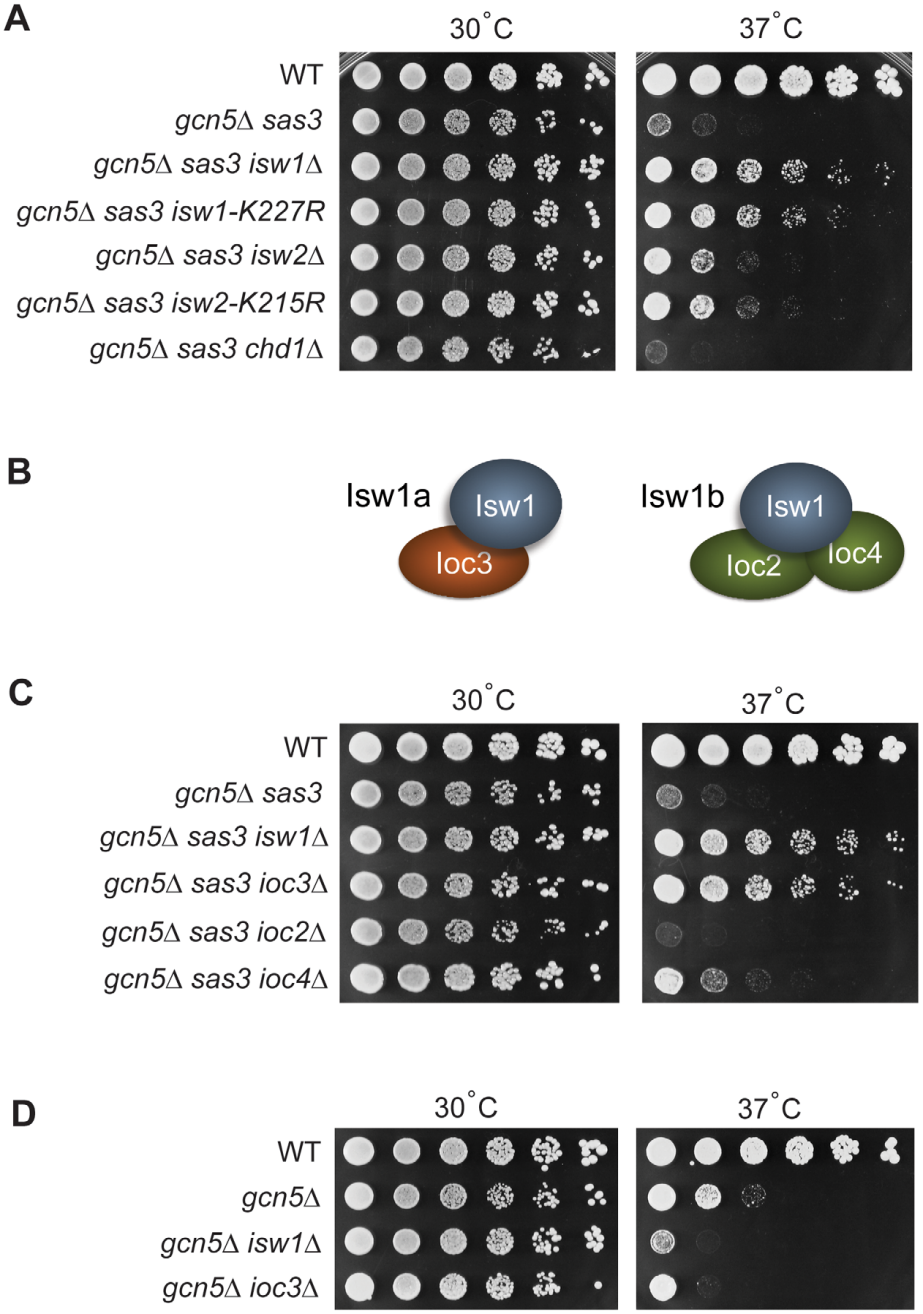
Sas3 and Gcn5 acetyltransferases and ISWI chromatin remodeling enzymes have antagonistic functions. (A) Inactivation of the chromatin remodeling ATPases Isw1 and Isw2 suppresses the growth defects of the *gcn5*Δ *sas3* mutant. Fivefold serial dilutions of cells were plated onto SC medium supplemented with 1 M sorbitol and grown for 4 days at the indicated temperatures (see also Figure S1A). (B) Isw1 is the catalytic subunit of two structurally distinct complexes. (C) Inactivation of the Isw1a complex (Isw1/Ioc3) suppresses the *gcn5*Δ *sas3* mutant. Strains were grown and plated as in panel A. (D) Inactivation of the Isw1a complex (Isw1/Ioc3) does not rescue the temperature sensitivity of the *gcn5*Δ mutant. Strains were plated on SC and grown for 4 days.

First, the temperature-sensitive phenotype of the *gcn5*Δ *sas3* double mutant was exacerbated upon deletion of *CHD1* (Figure 1A and Figure S1A). This suggested parallel functions, likely through overlap in transcriptional regulation, in agreement with previous studies [12], [17]. Second, and in distinct contrast, deletion of *ISW1* and to a lesser extent *ISW2*, improved growth of the *gcn5*Δ *sas3* cells (Figure 1A). Deletion of *ISW2* does not further restore growth of the *gcn5*Δ *sas3 isw1Δ* mutant, indicating that rescue is maximal upon inactivation of Isw1 (Figure S1B).

The Isw1 and Isw2 chromatin remodeling complexes use the energy of ATP hydrolysis to alter nucleosome positioning [32]–[35]. To determine if the ATP-dependent catalytic activity, and not some other property of the enzymes was responsible for suppression, we analyzed the *isw1*-*K227R* and *isw2*-*K215R* mutants that affect ATP-binding sites to inactivate the enzymes [32]. Figure 1A shows that both catalytic mutations rescued the temperature sensitivity of the *gcn5*Δ *sas3* mutant, with *isw1-K227R* having the stronger effect. Thus, suppression is dependent on the catalytic activities of the ISWI ATPases.

The suppression observed was unexpected since most reported interactions between chromatin modifying enzymes and chromatin remodelers describe parallel functions, often through recognition of modified nucleosomes by the remodelers [12], [17], [18], [36]–[38]. Because inactivation of the ATPase function of Isw1 consistently resulted in a stronger rescue phenotype than that of Isw2, we focused on dissecting the mechanism underlying the potential antagonism between *ISW1* remodeling and acetyltransferase activities mediated by *SAS3* and *GCN5*.

### The Sas3 and Gcn5 acetyltransferases counteract Isw1 function through the Isw1a complex

The Isw1 ATPase is the catalytic subunit of two distinct complexes (Figure 1B and reviewed in [1], [39]). The Isw1a complex includes the non-catalytic subunit Ioc3, whereas the non-catalytic subunits of Isw1b are Ioc2 and Ioc4 [32], [35]. The *in vivo* functions of these two complexes are not yet fully established. Microarray studies reveal that the Isw1a and Isw1b complexes have overlapping roles in transcriptional regulation at some genes, but distinct functions at others [35]. To determine whether one or both Isw1 complexes are involved in antagonizing Sas3 and Gcn5 function, we inactivated the complexes individually by deleting genes encoding their non-catalytic subunits. The *ioc3Δ* mutant, but neither *ioc2Δ* nor *ioc4Δ* strains, strongly rescued the *gcn5*Δ *sas3* phenotype, demonstrating that inactivation of the Isw1a complex is primarily responsible for the suppression mediated by loss of Isw1 (Figure 1C). Furthermore, deletion of *IOC2* exacerbated loss of viability, whereas *ioc4Δ* had no significant reproducible effects. This suggests an additional relationship between the Isw1b complex and the acetyltransferases Sas3 and Gcn5, and further supports the existence of distinct functions for the two Isw1 complexes. Since combined inactivation of both complexes through deletion of *ISW1* rescued the temperature sensitivity, Isw1a appears to have a prominent role in antagonizing Sas3 and Gcn5 activities.

Previous studies demonstrated interactions between the SWI-SNF remodelers and Gcn5 alone, independent of Sas3 [11], [12]. To determine if the antagonism between Sas3 and Gcn5 and the Isw1a complex is Gcn5-specific or acts through shared Sas3 and Gcn5 functions, we asked if inactivation of the Isw1a complex rescued temperature sensitivity associated with the single *gcn5*Δ mutation. Deleting *IOC3* or *ISW1* did not rescue, demonstrating that these acetyltransferases counteract Isw1a through shared functions of both acetyltransferases (Figure 1D).

### Loss of Sas3 and Gcn5 acetyltransferases modestly alters Isw1a recruitment at some active genes

**Figure 2:**
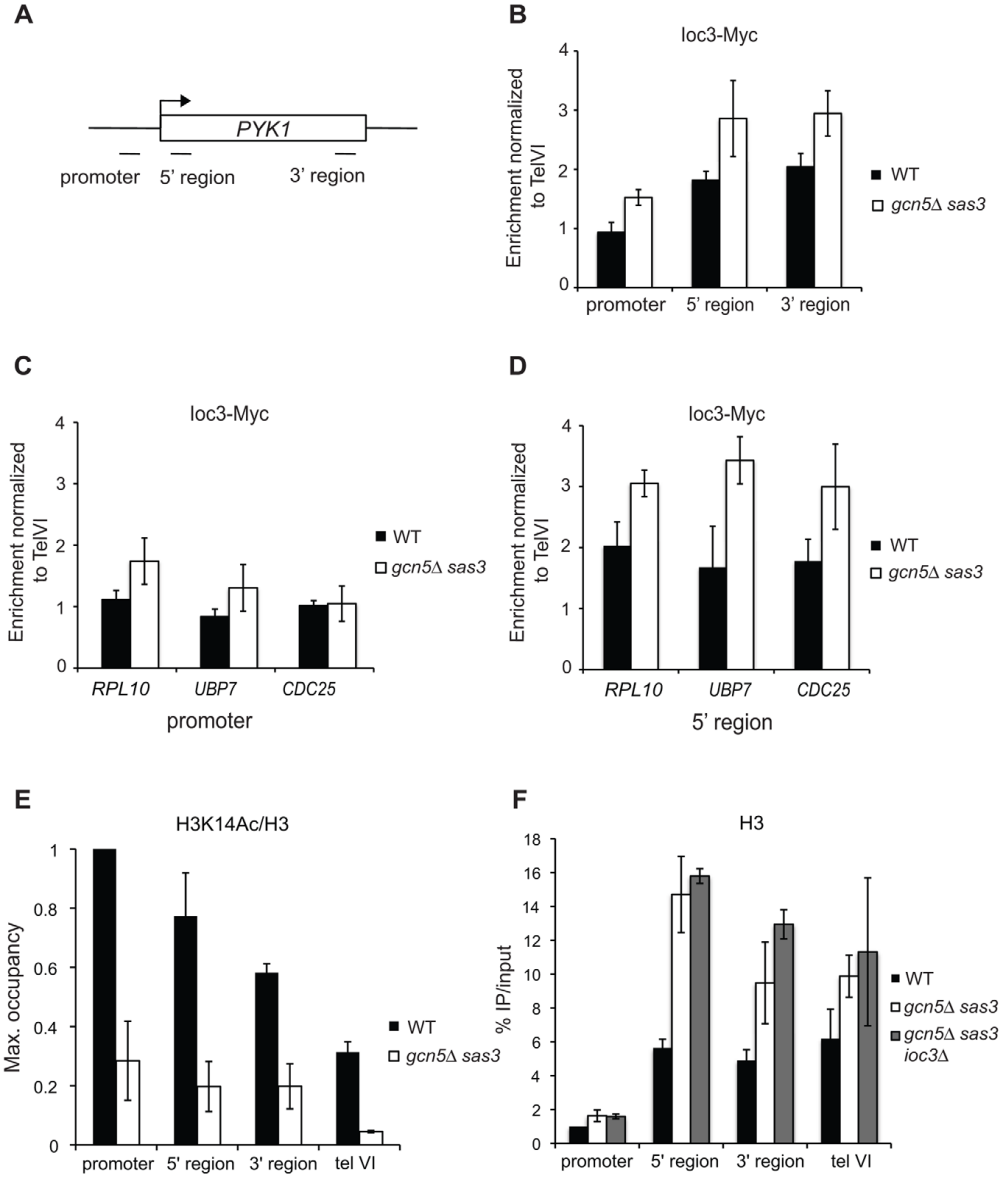
Loss of the Sas3 and Gcn5 H3 acetyltransferases modestly alters Isw1a recruitment to chromatin. (A) Schematic representation of the *PYK1* locus and positions of primer pairs used for ChIP analysis in (B), (E) and (F). Ioc3-Myc occupancy was assayed by ChIP in wild-type and *gcn5*Δ *sas3* cells grown in SC medium at 34°C over the *PYK1* gene (B), at promoter (C) and 5′ coding regions (D) of *RPL10*, *CDC25* and *UBP7* genes. (E) Histone H3K14 acetylation occupancy was analyzed by ChIP over the *PYK1* gene in wild-type and *gcn5*Δ *sas3* cells grown in SC medium at 34°C. (F) Histone H3 occupancy over the *PYK1* gene was assayed by ChIP in wild-type, *gcn5*Δ *sas3*, and *gcn5*Δ *sas3 ioc3Δ* cells grown in SC medium at 34°C. Ioc3-Myc occupancy was normalized to the telVI control region. H3K14Ac and H3 levels were normalized to the promoter region of *PYK1*. The values represent the means from two or more independent experiments, with error bars reflecting standard deviations.

Isw1 contains a SANT domain (reviewed in [40]) that is critical for binding to chromatin at regulated genes *in vivo* [41]. Biochemical studies indicate that the SANT domains from Ada2 and SMRT preferentially bind unacetylated histone H3 tails [42], [43]. To determine whether H3 acetylation antagonizes Isw1 recruitment to chromatin, we evaluated Ioc3-Myc occupancy in *gcn5*Δ *sas3* cells by chromatin immunoprecipitation (ChIP) at transcriptionally active target genes (Figure 2). Indeed, Sas3 and Gcn5 acetyltransferases are recruited to a similar set of actively transcribed genes, which correlate with H3K14 acetylation [26]. We selected the *PYK1* gene for analysis since H3 acetyltransferases and Isw1 are enriched at this locus [24], [26], [44]. For other candidate genes, a recent genome-wide study revealed that Sas3 is enriched at *RPL10* whereas *UBP7* and *CDC25* are impaired for H3K14 acetylation in a *sas3Δ* strain [26].

We observed that Ioc3 occupancy is enriched in the coding region compared to the promoter at the *PYK1*, *RPL10*, *UBP7* and *CDC25* genes (Figure 2B, Figure 2C and Figure 2E), as previously described for Isw1 at regulated genes [41]. The inactivation of Sas3 and Gcn5 modestly increased the recruitment of Ioc3 at promoter and coding regions of *PYK1* (Figure 2B), although the levels of H3K14 acetylation were severely decreased at this locus (Figure 2E). Similarly, Ioc3 occupancy was moderately affected by loss of Sas3 and Gcn5 at the promoter of *RPL10* and at coding regions of *RPL10*, *UBP7* and *CDC25* (Figure 2C and Figure 2D). Of note, nucleosome density increased in the coding region of *PYK1* upon inactivation of Sas3 and Gcn5, as revealed by H3 occupancy (Figure 2F).

### Inactivation of the Isw1a complex does not rescue defects in H3 acetylation, nor nucleosome occupancy

As the combined loss of Sas3 and Gcn5 resulted in a dramatic loss of H3 acetylation, we asked if suppression mediated by inactivation of the Isw1a complex might rescue this defect. Although the chromatin remodeling ATPase Isw1 has not been reported to regulate histone H3 acetylation, we hypothesized that nucleosome repositioning resulting from Isw1a inactivation might rescue *gcn5*Δ *sas3* defects. Since K14 is the major and common target of Sas3 and Gcn5 acetyltransferases *in vitro* and *in vivo* [22], [26], [45], we assayed global levels of H3K14 acetylation in wild-type, *gcn5*Δ *sas3* and *gcn5*Δ *sas3 ioc3Δ* cells by protein immunoblotting. As previously described [22], H3K14 acetylation decreased in the *gcn5*Δ *sas3* strain, however there was no significant difference in acetylation in the *gcn5*Δ *sas3 ioc3Δ* strain (Figure S3).

**Figure 3:**
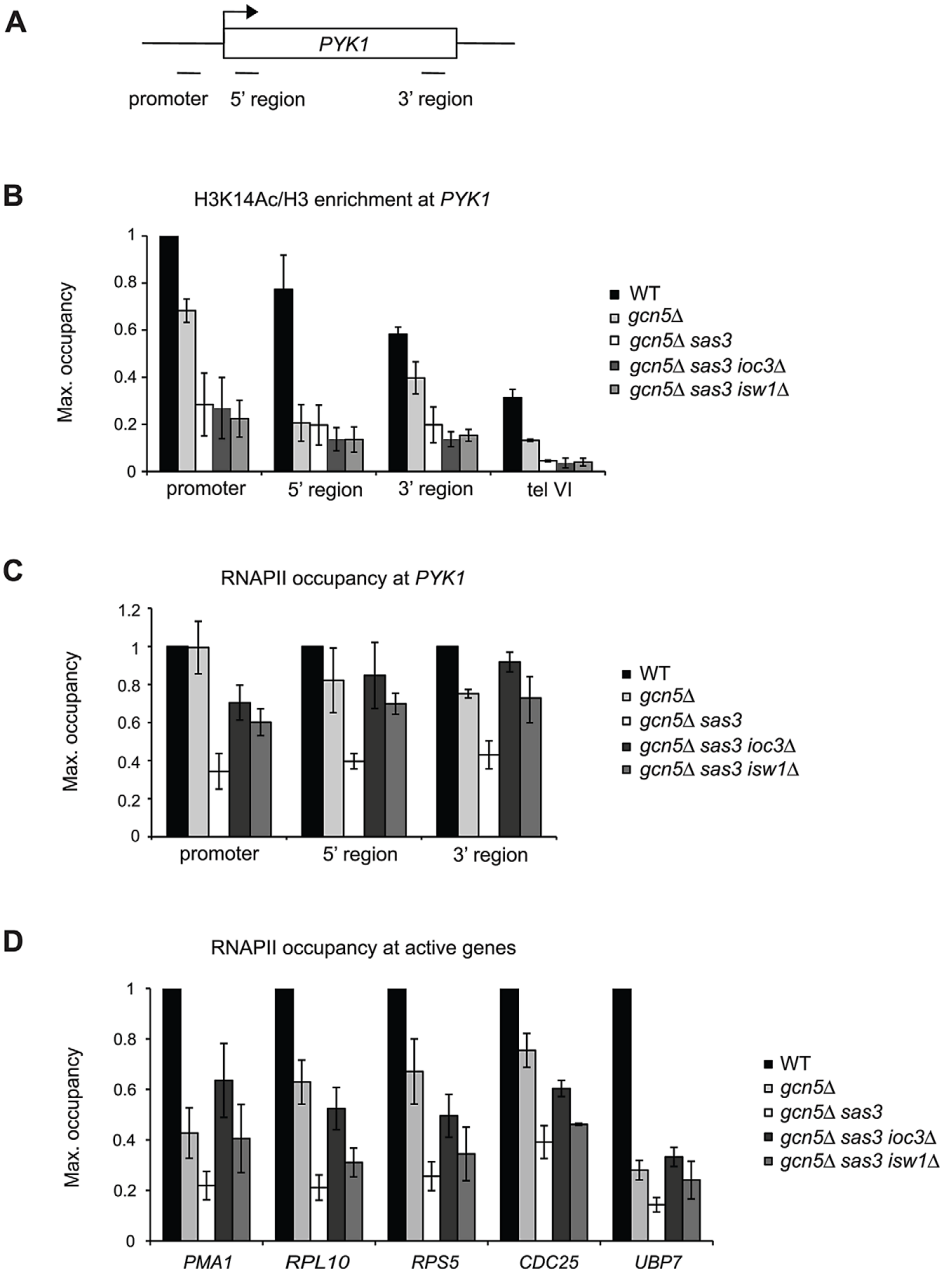
Sas3 and Gcn5 acetyltransferases and Isw1a antagonistically regulate RNAPII recruitment to active genes. (A) Schematic representation of the *PYK1* locus and positions of primer pairs used for ChIP analysis in (B) and (C). (B) Histone H3K14 acetylation (Ac) levels and (C) RNAPII occupancy over the *PYK1* gene were assayed by ChIP analysis of cells grown in SC medium at 34°C. H3K14Ac ChIP samples were normalized to total H3. (D) RNAPII occupancy was analyzed by ChIP as in (C), but at the 5′ regions of *PMA1, RPL10*, *RPS5*, *CDC25* and *UBP7* genes. H3K14Ac levels were further normalized to the promoter region of *PYK1*, and RNAPII occupancies were normalized to WT for each locus, arbitrarily set to 1. The values represent the means from two or more independent experiments, with error bars reflecting standard deviations.

Because global restoration of H3K14 acetylation did not occur, we tested the idea that locus-specific changes might be responsible for rescue of the *gcn5*Δ *sas3* mutant by assaying the local levels of H3K14 acetylation at the Isw1-responsive *PYK1* gene under suppressing conditions. H3K14 acetylation levels were impaired over the whole *PYK1* gene (promoter and coding regions) in the *gcn5*Δ strain, and more dramatically in the *gcn5*Δ *sas3* strain (Figure 3B), demonstrating that Sas3 and Gcn5 are responsible for H3K14 acetylation at this locus. Further, as revealed by the levels of H3K14 acetylation in the *gcn5*Δ strain, Sas3 markedly contributed to H3K14 acetylation in the promoter and 3′ regions of the *PYK1* gene (Figure 3B). Yet, no further difference in H3K14 acetylation levels was observed upon deletion of *IOC3* or *ISW1* (Figure 3B). Similarly, the elevated H3 occupancy observed in the *gcn5*Δ *sas3* strain at the *PYK1* gene remained unaffected by inactivation of Ioc3 (Figure 2F). Because inactivation of Isw1a does not suppress Sas3 and Gcn5 defects by directly restoring K14 acetylation either globally or locally, or by decreasing nucleosome occupancy, suppression must occur through some other mechanism.

### Isw1a and the acetyltransferases Sas3 and Gcn5 regulate the recruitment of RNAPII to active genes

We asked if the interaction between Isw1a and H3 HATs is related to transcription as reflected by RNAPII occupancy. Indeed, genome-wide analyses revealed that Sas3 and Gcn5 are recruited to actively transcribed genes and that their occupancies correlate with transcriptional rates [20], [24], [26]. Based on the fact that Isw1 also regulates transcriptional activation [35], [44], we assayed RNAPII occupancy at the *PYK1* gene.

In agreement with the proposed roles of Sas3 and Gcn5 in transcriptional activation, we observed that loss of these HATs resulted in defective RNAPII recruitment at *PYK1* (Figure 3C and Figure S4A). Loss of Gcn5 slightly impaired occupancy at the 3′ region yet did not affect the promoter and 5′ regions, highlighting a role for Sas3 in RNAPII recruitment and in transcriptional activation. Significantly, deletion of *IOC3* and *ISW1* in a *gcn5*Δ *sas3* mutant partially rescued RNAPII occupancy at the promoter and coding region of *PYK1* (Figure 3C and Figure S4A).

We evaluated RNAPII at the other active genes described above to determine how general the suppressive effects were on occupancy. For this study, we also included the additional Isw1 and Sas3 target genes *PMA1* and *RPS5*, respectively [26], [44]. As shown for *PYK1*, we found that Sas3 and Gcn5 contributed to the recruitment of RNAPII at *PMA1*, *RPL10*, *RPS5*, *CDC25* and *UBP7* coding regions (Figure 3D and Figure S4B). Furthermore, deletion of *IOC3* improved RNAPII occupancy at these genes, more than the modest effects observed with *isw1Δ*. We asked if these differences in the recruitment of RNAPII influenced trancription (Figure S4C). We assayed the steady state levels of *PYK1, PMA1* and *RPL10* mRNAs by RT-qPCR, and observed no significant changes in the *gcn5*Δ *sas3* and *gcn5*Δ *sas3 ioc3Δ* mutants when compared to the wild-type strain (Figure S4C). Together these results demonstrate that Sas3 and Gcn5 acetyltransferases and the Isw1a complex have antagonistic roles in chromatin as reflected by recruitment of RNAPII.

### Isw1-dependent rescue of RNAPII at *PYK1* is independent of nucleosome repositioning

RNAPII function and regulated recruitment during transcription are critically dependent on chromatin architecture. Given that Sas3 and Gcn5 acetyltransferases and the Isw1a complex have opposing effects on RNAPII recruitment to transcriptional target genes, we asked whether they also antagonistically regulate nucleosomal occupancy at the *PYK1* locus. It has been suggested that *PYK1* chromatin structure is dependent on Isw1 since the ATPase is associated with this coding region [44].

**Figure 4:**
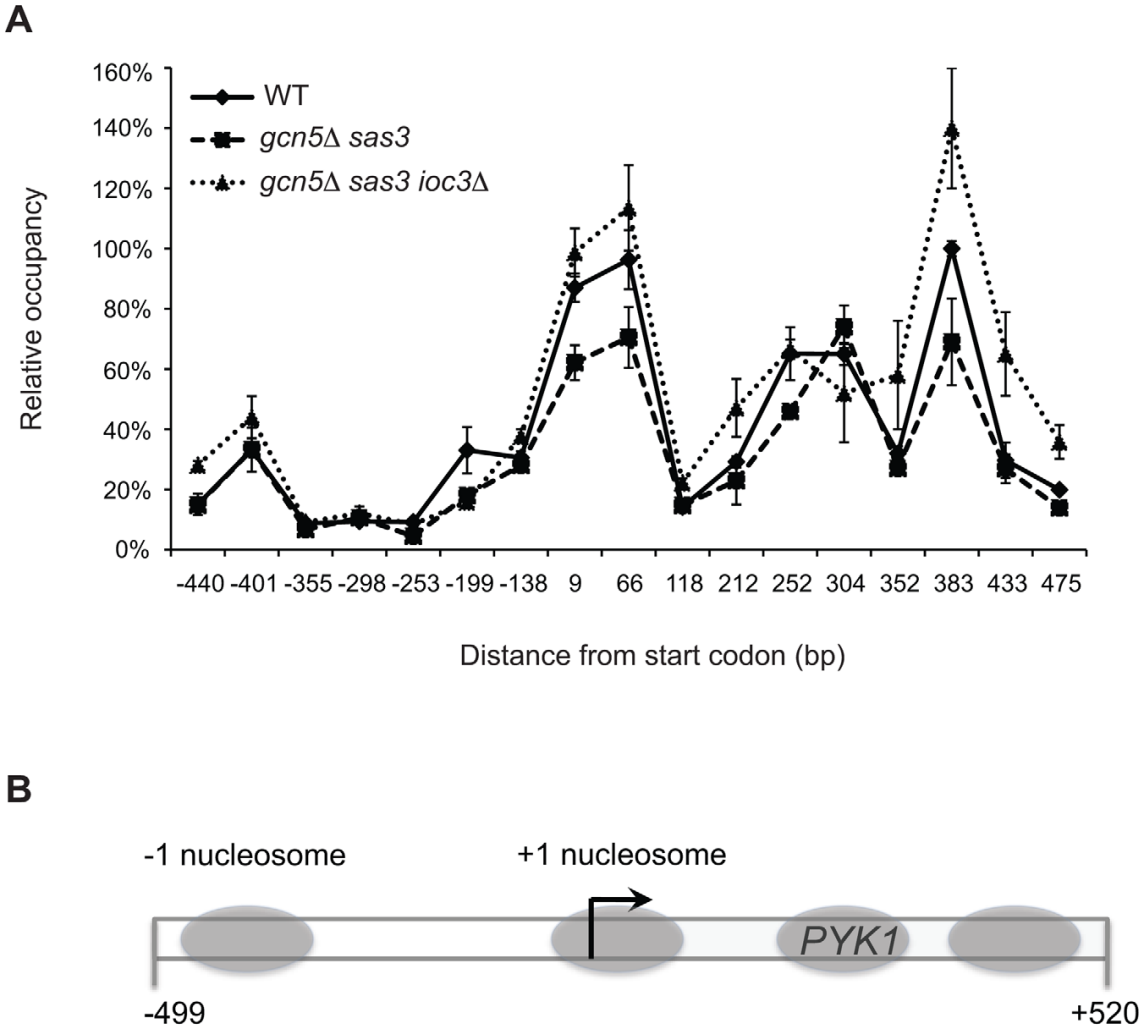
Inactivation of the Isw1a complex in the *gcn5*Δ *sas3* mutant does not significantly alter nucleosome positioning at the *PYK1* gene. (A) A nucleosome scanning assay was performed on chromatin from WT, *gcn5*Δ *sas3*, and *gcn5*Δ *sas3 ioc3Δ* cells grown in SC medium at 34°C. Mononucleosomal DNA was purified and analyzed by real-time qPCR using seventeen overlapping primer pairs spanning the promoter and 5′ coding region of *PYK1*. Values are expressed as percentage of input, and normalized to the *PHO5* TATA region [77]. The values represent the means from three independent experiments, with error bars reflecting standard deviations. (B) Diagram of the *PYK1* locus indicates the positions of nucleosomes (gray ovals) extrapolated from the MNase protection assay.

We examined nucleosomal organization at *PYK1* by comparing micrococcal nuclease (MNase) cleavage patterns of chromatin prepared from wild-type and mutant strains (Figure S5). We observed no significant differences in MNase cleavage patterns in *gcn5*Δ *sas3 ioc3Δ* cells when compared to *gcn5*Δ *sas3* or wild-type cells (Figure S5). One possible explanation was that the MNase assay might not detect the Isw1 remodeling activities at the *PYK1* gene. For example, the ISWI-dependent rescue may involve nucleosome repositioning at a level too subtle to be detected using the MNase mapping assay. Indeed, Isw1 and Isw2 remodeling activities increase genome-wide nucleosome occupancy at mid-coding regions and intergenic regions, respectively, to prevent cryptic transcription [46], [47]. In order to map nucleosome location with high resolution at the *PYK1* gene, we took advantage of nucleosome-scanning analysis [48]. This method couples isolation of mononucleosomal DNA by MNase digestion with qPCR analysis using a set of overlapping primer pairs spanning the region of interest [48]. Nucleosome scanning analysis of the *PYK1* region revealed the presence of four positioned nucleosomes, one located in the promoter region and three others positioned in the 5′ coding region (Figure 4). Further, a 150 bp region highly sensitive to MNase digestion has been identified in the promoter region (−350 to −200 from the start codon), indicating the presence of a nucleosome depleted region (NDR) (Figure 4). This organization is characteristic of “open” promoters which favor the binding of transcription factors at the expense of nucleosomes [49]. Open promoters are a common property of constitutive genes, such as the conditionally essential gene *PYK1*. No major changes in nucleosome positioning were observed upon inactivation of Gcn5 and Sas3, or further depletion of Ioc3 (Figure 4).

### A dual role for the ATPase Isw1 revealed by functional interactions between the Isw1a and Isw1b complexes and the acetyltransferases Sas3 and Gcn5

Multiprotein complexes can be altered both by mutation and by changing subunit abundance through gene dosage. We took advantage of gene overexpression (reviewed in [50]–[52]) as an independent approach to probe the relationship of *ISW1* to *SAS3* and *GCN5*.

**Figure 5:**
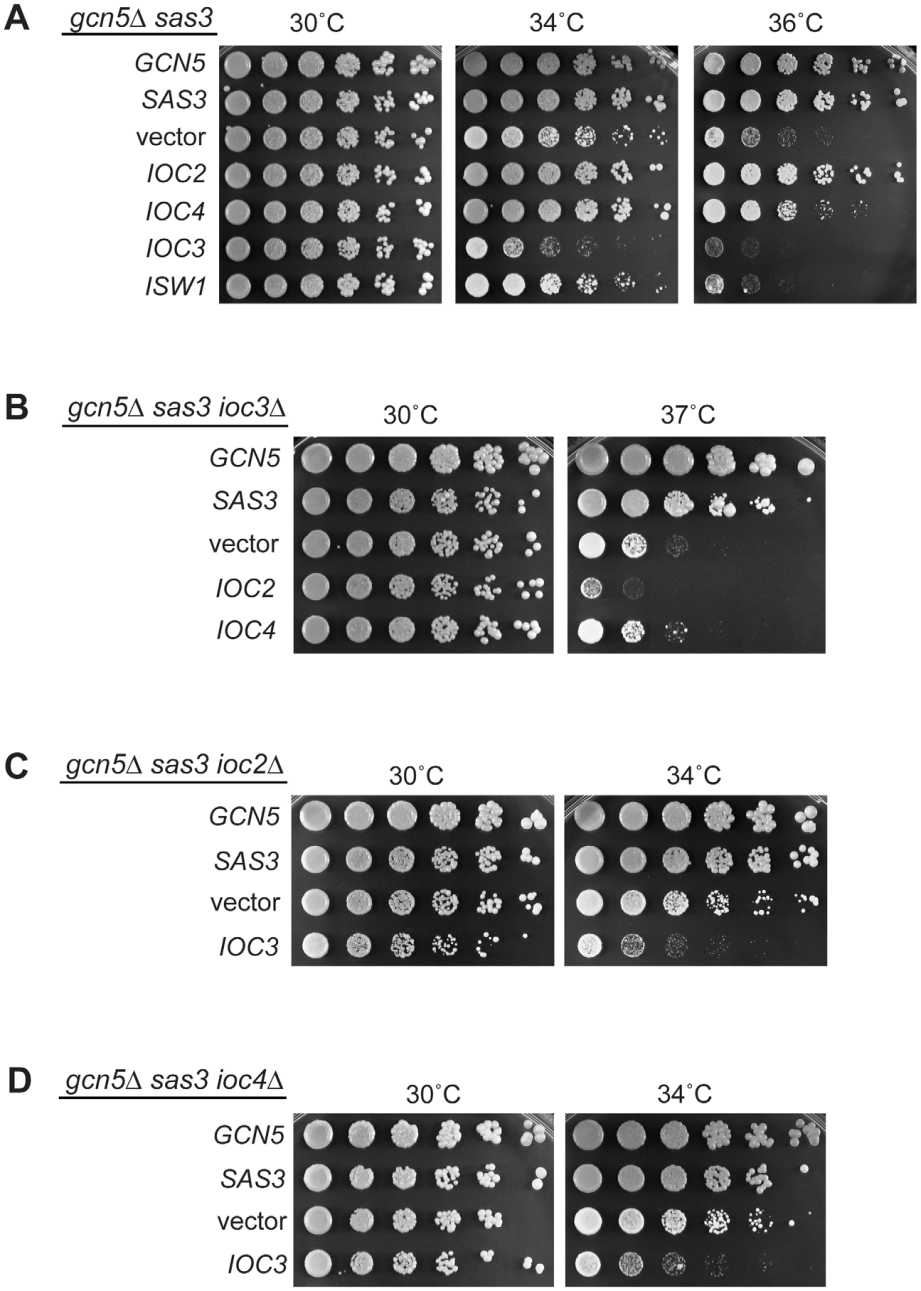
Functional interactions between Isw1a/b complexes and acetyltransferases reveal a dual role for the ATPase Isw1. (A) Overexpression of the Isw1b complex (Isw1/Ioc2/Ioc4) rescues the temperature sensitivity of the *gcn5*Δ *sas3* mutant. In contrast, overexpression of the Isw1a complex (Isw1/Ioc3) exacerbates the growth defects of the HAT mutants. The *gcn5*Δ *sas3* mutant was transformed with 2μ-*LEU2* plasmids containing *GCN5*, *SAS3*, vector, *IOC2*, *IOC4*, *IOC3* and *ISW1*. Transformants were plated onto SC–Leu medium and grown for 4 days. (B) Suppression of temperature sensitivity mediated by overexpression of the Isw1a complex (Isw1/Ioc2/Ioc4) requires a functional Isw1b complex (Isw1/Ioc3). The *gcn5*Δ *sas3 ioc3Δ* mutant was transformed with the indicated plasmids. Suppression was assayed by growth on SC–Leu medium. Note that the growth difference between the *gcn5*Δ *sas3 ioc3Δ* mutant in Figure 1C and the vector control shown here is due to the presence of sorbitol in the medium in Figure 1C, which partially relieves cell growth defects at elevated temperatures. Plating is shown here without sorbitol to increase the dynamic range of the assay. (C) and (D) Exacerbation of the temperature sensitivity mediated by overexpression of the Isw1b complex (Isw1/Ioc3) is not dependent on a functional Isw1a complex (Isw1/Ioc2/Ioc4). Transformants of the *gcn5*Δ *sas3 ioc2Δ* (C) and *gcn5*Δ *sas3 ioc4Δ* (D) mutants were assayed on SC–Leu medium.

Increased gene dosage of *IOC2* or *IOC4* restored viability at elevated temperature, whereas overexpression of *IOC3* exacerbated sickness of the *gcn5*Δ *sas3* mutant (Figure 5A). Increased *ISW1* also interfered with growth, confirming its generally antagonistic function. Furthermore, increased gene dosage of *IOC2* did not rescue thermosensitivity of the *gcn5*Δ single mutant (Figure S6A), nor did it serve as a bypass suppressor of the *gcn5*Δ *sas3Δ* strain. Together these results suggest that increasing the stoichiometry of the Isw1b complex can ameliorate the negative effects of the Isw1a complex in the *gcn5*Δ *sas3* mutant.

To determine if the Isw1a and Isw1b complexes act in parallel or in the same pathway, we performed a series of analyses to dissect the relative contribution of each complex. We first evaluated increased gene dosage of the Isw1b complex components in a strain depleted for the Isw1a complex. Of note, deletion of *IOC3* partially rescues the *gcn5*Δ *sas3* temperature sensitivity. Therefore, although the *gcn5*Δ *sas3 ioc3Δ* strain is less sensitive than the *gcn5*Δ *sas3* (Figure 1C), this mutant is still somewhat temperature sensitive (Figure 5B), and provides the possibility for a dynamic range in which growth enhancement or inhibition could be observed. We found that overexpression of *IOC2* or *IOC4* did not rescue the residual *gcn5*Δ *sas3* temperature sensitivity in an *ioc3Δ* background, demonstrating that the Isw1a complex is required for Isw1b-mediated dosage suppression (Figure 5B).

We next evaluated whether Isw1a overexpression enhanced the *gcn5*Δ *sas3* phenotype in a strain depleted for Isw1b by deleting *IOC2* and *IOC4*. Overexpression of *IOC3* still exacerbated the *gcn5*Δ *sas3* temperature sensitivity in a strain depleted for Isw1b (Figure 5C and Figure 5D). These results support the idea that increasing the relative balance of the Isw1b complex suppresses the *gcn5*Δ *sas3* phenotype by counteracting Isw1a function. In addition, we observed that deletion of *ISW1* interfered with the phenotypes resulting from overexpression of the *IOC* genes in the *gcn5*Δ *sas3* strain, underscoring the critical role for the Isw1 catalytic ATPase itself (Figure S6B).

## Discussion

Since the acetyltransferase activities of Sas3 and Gcn5 were initially reported to be essential [22], understanding the molecular mechanisms underlying this synthetic lethality has remained incomplete. Indeed, whereas disruption of these H3-specific acetyltransferases results in cell death, mutation of lysine residues in histone H3 that are known to be targeted by Sas3 and Gcn5 has only modest phenotypes [27], [28]. This discrepancy and the growing number of validated non-histone acetylation targets [53], [54] strongly suggest that the Sas3-Gcn5 essential function may reside in acetylation of histone and/or non-histone targets.

Our previous work demonstrated that the Sas3 and Gcn5 acetyltransferases are jointly required for viability and are responsible for the majority of histone H3 acetylation at K9 and K14 *in vivo* [22]. Here we show that both HATs contribute to *in vivo* acetylation of H3K14 at an actively transcribed gene, and furthermore we correlate the dramatic decrease in H3K14 acetylation with defective recruitment of RNAPII to promoter and coding regions. This reinforces the view that H3K14 acetylation is an epigenetic mark associated with transcriptional activation [20]. Moreover, the joint contributions of Sas3 and Gcn5 provide a molecular explanation for previous results showing that loss of Gcn5 only modestly affects acetylation of H3K14 at various genes [23], [55].

We found that inactivation of ISWI family remodelers alleviates *gcn5*Δ *sas3* cell death. In deep contrast, inactivation of the Chd1 remodeler exacerbates the sickness of the *gcn5*Δ *sas3* strain. Unlike other chromatin remodeler families, most ISWI complexes are required for formation of repressed structures [32], [44], [46], [56], [57] and chromosome compaction *in vivo* [58]–[60]. Furthermore, inactivation of the linker histone H1, another critical player in chromatin condensation, also rescues the growth defect of a *gcn5*Δ *sas3* mutant [28]. Thus, destabilizing chromatin compaction, repressed structures, or higher-order chromatin structures through inactivation of ISWI complexes or histone H1 partially relieves growth defects in *gcn5*Δ *sas3* cells. This supports the view that Sas3 and Gcn5 acetyltransferase activities counterbalance negative effects of repressed structures and condensed chromatin. In agreement with this model, we observed that inactivation of the Isw1a complex rescues RNAPII recruitment at active genes in a *gcn5*Δ *sas3* mutant, to levels similar to those observed in the *gcn5*Δ mutant. This correlates with the degree of rescue observed for cell viability, strongly suggesting that Isw1-dependent rescue is mediated at least partially through the recruitment of RNAPII at actively transcribed genes. Similarly, the H4 HAT Esa1 can overcome the repressing function of Isw1 on transcription [61]. Conditional inactivation of Esa1 leads to defects in RNAPII recruitment at the *MET16* gene upon induction, as well as impaired accumulation of *MET16* transcript. Deletion of *ISW1* was also seen to restore *MET16* RNA levels and RNAPII distribution, which is very similar to our observations with RNAPII recruitment at active genes in the *gcn5*Δ *sas3* mutant. However, the outcomes of these genetic interactions are clearly different: whereas inactivation of Isw1 rescues the sickness of *gcn5*Δ *sas3* cells, it exacerbates cell growth defects of *esa1* mutants [62], [63]. These differences are likely to reflect the distinct biological functions and substrates of the Sas3/Gcn5 and Esa1 acetyltransferases.

In contrast with Sas3 and Gcn5 acetyltransferases, functional characterization of ISWI enzymes in transcriptional regulation reveals significant roles in gene repression [35], [46], [47], [56]. Future studies should determine if Sas3 and Gcn5 acetyltransferases and ISWI family remodelers act in the same pathway.

In Drosophila, H4K16 acetylation regulates chromatin compaction by reducing the ability of ISWI to bind chromatin [64], [65]. Such a mechanism has not been described for H4 acetylation in yeast, and we show here that loss of the main H3 acetyltrasferases Sas3 and Gcn5 only modestly affects Isw1a recruitment to chromatin at some active genes (Figure 2). Thus it appears that Sas3 and Gcn5 acetyltransferase activities may counteract Isw1 function independently of chromatin binding. Such regulation has been reported for the Chd1 and Isw2 remodeling enzymes: H4 acetylation antagonizes nucleosome remodeling by lowering the catalytic turnover of ATP hydrolysis without affecting nucleosome binding [38]. Further, Sas3 and Gcn5 acetyltransferases might control Isw1 function directly through acetylation. In *Drosophila*, Gcn5 acetylates Isw1 at a single lysine *in vitro* and *in vivo* [66]. This acetylation occurs in a region similar to the N-terminal tail of H3, at a residue corresponding to K14. We also detected low levels of Isw1 acetylation in wild-type cells, with a two-fold decrease in the *gcn5*Δ *sas3* mutant (data not shown).

Although loss of the ATP-dependent remodeling activity of Isw1 is required to restore cell viability, we observed no significant effects on nucleosome positioning upon inactivation of Isw1a at the *PYK1* gene. Of note, nucleosome occupancy appears slightly reduced in the *gcn5*Δ *sas3* mutant, at the predicted nucleosomes +1 and +3 (Figure 4). This appears in contrast with the increased H3 occupancy observed at the *PYK1* promoter and coding region by ChIP (Figure 2F). However, it should be noted that nucleosome occupancy assayed by the MNase scanning method was normalized to the *PHO5* promoter while the ChIPs for H3 were not, which is likely to account for this discrepancy. Alternatively, these results may suggest a local change in nucleosome assembly or disassembly in *gcn5*Δ *sas3* mutants that results in accumulation of incomplete nucleosomes. Addition and removal of the H3/H4 tetramer is the first and last step of nucleosome assembly and disassembly, respectively (reviewed in [67]). Tetrasomes protect around 80 base pairs of DNA, below the resolution of our MNase-qPCR study, which may contribute to the loss of nucleosome signal in the MNase assay. This possibility can be explored in future studies by evaluating H2B occupancy at the 5′ region of *PYK1* and by testing genetic interactions between *gcn5*Δ *sas3* and histone chaperones.

An additional possibility for the ISWI-dependent chromatin remodeling rescue in *gcn5*Δ *sas3* mutants is that it may be mediated through alteration of higher order chromatin structures. Indeed, the *Drosophila* ISWI-containing remodeler ACF can assemble regularly spaced arrays of H1-containing nucleosomes and can further catalyze repositioning of chromatosomes (nucleosome+H1) in chromatin fibers [68], [69]. Furthermore, inactivation of histone H1 also rescues the growth defect of a *gcn5*Δ *sas3* mutant [28]. Therefore, restoration of RNAPII upon inactivation of Isw1a complex might rely on different states of chromatin that differ in the periodicity of chromatosome arrays.

An unexpected finding uncovered by our genetic analysis is the opposing functions of Isw1a and Isw1b complexes in relation to the H3 HATs. Specifically, we observed that rescue mediated by Ioc2 and Ioc4 requires Ioc3, but this requirement is not reciprocal. This suggests that, at least in the context of *gcn5*Δ *sas3*, the Isw1b complex antagonizes the function of the Isw1a complex. Early on, functional characterization of these two complexes revealed distinct roles [35]. Notably they differ in their biochemical activities to bind and space nucleosomes [35], [70], [71], their nucleosome remodeling properties *in vivo* [46], [47], as well as their roles in transcriptional regulation [35], [44]. Similarly in other eukaryotes, ISWI complexes that share the same catalytic subunit have distinct biological functions, specified by their associated proteins (reviewed in [1], [39]). The observations reported here bring new understanding toward defining their differences by showing that one ISWI complex may counteract the function of another ISWI complex.

Together, the results from this study deepen understanding of the essential roles for H3 HATs. Not only do the HATs positively promote gene-specific transcriptional activation, as has been well established, they also have a critical role in balancing the activities of ATP-driven chromatin remodelers. The functional antagonism between Isw1a and the Sas3 and Gcn5 acetyltransferases further defines biological distinctions between the Isw1 enzyme's catalytic activities in its two structurally distinct complexes. Thus, in addition to interactions between histone modifications defining transcriptional output (reviewed in [6]–[8]), it is likely that future studies will reveal increasingly diverse interactions between the modifying machines and the complexes that dynamically define chromatin architecture through its remodeling.

## Materials and Methods

### Yeast strains and plasmids

Strains used are listed in Table S1 and are in the W303 background. Gene deletions and other standard procedures were performed as described [72]. The *gcn5*Δ *sas3* conditional mutant was constructed with a chromosomal allele of the *sas3*-*C357Y, P375A* double point mutation. As described for the plasmid conditional mutant [22], the chromosomal version of the mutant grows well at 30°C, but dies at 37°C. All strains carrying *isw1Δ::kanMX*, *ioc2Δ::kanMX*, *ioc3Δ::natMX*, *ioc4Δ::hphMX*, *isw1-K227R* and *isw2-K215R-3FLAG-kanMX* alleles were derived from the strains YTT441, YTT823, YTT825, YTT855 [35], YTT1223 and YTT1996 respectively, generous gifts from T. Tsukiyama. The *gcn5*Δ*::natMX* allele was obtained by marker swapping using p4339 (generous gift from C. Boone) on *gcn5*Δ*::kanMX*. The strains expressing Ioc3-13Myc and Isw1-13Myc from their chromosomal loci were constructed as described in [73]. All plasmids were derived from Yep351 (2μ *LEU2*). pLP645 was constructed by inserting a BamHI-SalI fragment containing *SAS3* into Yep351 opened with SalI and BamHI (J. Lowell). pLP1524 was obtained from a genomic library generously provided by S. Roeder [74]. It contains a 3.9 kb fragment encompassing *GCN5* gene (Chr.VII 995,188 to 998,784 bp). To construct Yep351-*IOC2* (pLP2234), a HindIII fragment (4.6 kb) containing *IOC2* was subcloned from pLP2170 (containing genomic fragment from Chr.XII 328,038 to 332,847 bp) into Yep351. To create Yep351-*ISW1* (pLP2256), a BamHI-PstI fragment was subcloned from pRS416-*ISW1* (a generous gift from T. Tsukiyama) into Yep351. Plasmid Yep351-*IOC4* (pLP2260) was constructed by PCR amplification of *IOC4* (−395 bp from start codon to +412 bp from stop codon), and cloned into Yep351 using HindIII-PstI restriction sites. Plasmid Yep351-*IOC3* (pLP2266) was constructed by PCR amplification of *IOC3* (−700 bp from start codon to +400 bp from stop codon), and cloned into Yep351 using HindIII-PstI restriction sites. Integrity of the constructs was confirmed by DNA sequencing.

### Temperature sensitivity assays

Cultures were grown for 2 days in SC or appropriate selective medium at permissive temperature. Cells were diluted to an A_600_ of 1 and plated in fivefold serial dilutions onto SC or selective medium, supplemented with 1 M sorbitol where indicated, and incubated for 4 days at the indicated temperatures prior to data collection.

### ChIP

Chromatin immunoprecipitation assays were performed as described previously [75] with minor modifications. Cultures were grown in SC medium at 34°C to A_600_ of 0.7–0.9 and cross-linked with 0.86% formaldehyde for 40 min. Immunoprecipitations (IP) were either pre-cleared with CL4B Sepharose beads (Sigma) for 1 hour at 4°C, then incubated overnight at 4°C with anti-RNAPII (8GW16, Covance) or directly incubated overnight at 4°C with antibodies against H3 (07-690, Upstate/Millipore), acetylated H3K14 (07-353, Upstate/Millipore), or the myc epitope (9E10). DNA was purified using PCR purification columns (Qiagen) and analyzed by real-time PCR (MJ Research Opticon2 system). Primer sequences are listed in Table S2. For quantification of ChIP samples, standard curves were generated for each set of primers, and DNA from IP and input samples was assayed for each strain in triplicate real-time PCR reactions. Each IP sample was normalized to the control IP (i.e. no epitope or no antibody) by subtraction, divided by the input sample, and expressed as percent of input (% IP/input). The % IP/input values for H3K14Ac were further normalized to % IP/input values for total H3. The % IP/input values for Ioc3-Myc and RNAPII were normalized to telVI and rDNA 5S control regions, respectively. Data represent averages from two or more independent experiments.

### Nucleosome scanning analysis

Extracts from MNase digestions were prepared as described [76], [77]. Briefly, cultures were grown in SC medium at 34°C to an A_600_ of 0.7–0.9. Then ∼2×10^9^ cells were harvested, washed in 1 ml sorbitol 1 M, resuspended in 1 ml of zymolyase solution (sorbitol 1.1 M, 20 mM KPO_4_, pH7, 0.5 mM CaCl_2_, β-mercaptoethanol 0.5 mM, zymolyase 100T 1 mg/ml) and incubated for 1.5 min at room temperature. Spheroplasts were then washed twice in 1 M sorbitol and gently resuspended in 1.6 ml of cold buffer A (1 M sorbitol, 50 mM NaCl, 10 mM Tris-HCl, pH 7.4, 5 mM MgCl_2_, 0.5 mM spermidine, 0.075% NP40 and 1 mM β-mercaptoethanol). The cell slurry was divided into 400 µl aliquots, and each was added to a microfuge tube containing the MNase (0, 60, 150 and 400 U/ml final concentrations) and incubated at 37°C for 4 minutes. The reaction was stopped by addition of 40 µl of stop buffer (250 mM EDTA, 5% SDS). DNA purification was performed as described in [76]. MNase digested DNA was run out on a 1.5% agarose gel and mononucleosome sized fragments were excised and purified using Qiagen's Gel Extraction kit. Purified mononucleosomes were analyzed by real-time PCR using the MJ Research Opticon2 system. Primer sequences are listed in Table S2. For quantification of MNase digested samples, standard curves were generated for each set of primers. Digested and input DNAs were assayed for each strain with each primer set in triplicate PCR reactions. MNase digested samples were divided by the input value for each primer set to generate percent of input. This was further normalized to the *PHO5* TATA region [78].

The protein immunoblotting, mRNA quantification, and chromatin analysis techniques used in Figures S2, S3, S4, S5 are described in Supporting Methods (Text S1).

## Supporting Information

Figure S1 Functional interactions between Sas3 and Gcn5 acetyltransferases and chromatin remodeling enzymes ISWI and Chd1. (A) Inactivation of the Chd1 chromatin remodeling enzyme exacerbates the growth defects of the *gcn5*Δ *sas3* mutant. Five-fold serial dilutions of cells were plated onto SC medium supplemented when indicated with 1 M sorbitol, and grown for 4 days at the indicated temperatures (see also Figure 1A). (B) Inactivation of the chromatin remodeling enzyme Isw2 does not further rescue the growth defects of the *gcn5*Δ *sas3 isw1Δ* mutant. Strains were plated onto SC medium supplemented with 1 M sorbitol, and grown at the indicated temperatures.
(TIF)

Figure S2 Overexpression of Isw1b subunits does not affect Ioc3 protein levels. Increased gene dosage of *IOC2* and *IOC4* does not affect *IOC3* expression. WT and *gcn5*Δ *sas3* cells expressing Ioc3-Myc were transformed with *IOC2* or *IOC4* in the 2 µm plasmid and grown at 37°C. Ioc3-Myc levels were determined by immunoblotting using anti-Myc, normalized using anti-tubulin and further normalized to empty vector control for relative quantification. Shown is a representative blot of three experiments.
(TIF)

Figure S3 Deletion of *IOC3* does not restore bulk levels of H3K14 acetylation in *gcn5*Δ *sas3* cells. Whole cell protein extracts from wild-type, *gcn5*Δ *sas3*, *gcn5*Δ *sas3 ioc3Δ* cells were immunoblotted with anti-H3K14ac, and anti-H3 as a control for histone levels. Quantification of H3K14Ac was normalized to H3 levels with WT level set to 1.
(EPS)

Figure S4 Sas3 and Gcn5 acetyltransferases and Isw1a antagonistically regulate RNAPII recruitment to active genes, but do not alter gene expression. RNAPII occupancy over the *PYK1* gene (A) or at the 5′ regions of *PMA1, RPL10*, *RPS5*, *CDC25* and *UBP7* genes (B) were assayed by ChIP analysis of cells grown in SC medium at 34°C. RNAPII occupancies in (A) and (B) were normalized to 5′ region of *PYK1* and the *PMA1* gene respectively, arbitrarily set to 1. The values represent the means from two or more independent experiments, with error bars reflecting standard deviations. (C) cDNAs from WT, *gcn5*Δ *sas3*, and *gcn5*Δ *sas3 ioc3Δ* cells grown at 34°C were analyzed by quantitative PCR. Expression values are relative to *ACT1* and normalized to WT. The values represent the means from three independent experiments, with error bars reflecting standard deviations.
(EPS)

Figure S5 Loss of H3 HATs does not lead to major changes in nucleosome positioning at the *PYK1* gene. MNase analysis of *PYK1*-specific chromatin in wild-type, *gcn5*Δ *sas3* and *gcn5*Δ *sas3 ioc3Δ* cells. Chromatin was probed for *PYK1* following digestion with MNase at concentrations of 0, 60, 150 and 400 U/ml, and EcoRI digestion. Marker restriction digests (Marker) are positioned relative to schematic maps of the *PYK1* gene. Restriction site positions are relative to the transcriptional start site of *PYK1*. The black line corresponds to the probe used for indirect end labeling. Densitometric scans of chromatin digested with 60 and 150 U/ml of MNase for the wild-type and mutant strains, respectively, were generated using ImageJ software (National Institutes of Health, USA).
(EPS)

Figure S6 Requirements of Isw1 complex subunits for restoration of *gcn5*Δ and *gcn5*Δ *sas3* growth defects. (A) Increased gene dosage of *IOC2* did not rescue the temperature sensitivity of the *gcn5*Δ mutant. The *gcn5*Δ mutant was transformed with the indicated plasmids. Strains were plated on SC–Leu medium and grown for 4 days. (B) Suppression and exacerbation of temperature sensitivity mediated by overexpression of Isw1a and Isw1b complexes, respectively, require the Isw1 ATPase. The *gcn5*Δ *sas3 isw1Δ* mutant was transformed with the indicated plasmids. Transformed strains were plated onto SC–Leu medium and grown for 4 days at the indicated temperatures.
(EPS)

Table S1 Yeast strains used in this study.
(DOC)

Table S2 ChIP primers used in this study.
(DOCX)

Text S1 Supporting Methods.
(DOCX)
