# Supplementary figures and images for: Functional Antagonism between Sas3 and Gcn5 Acetyltransferases and ISWI Chromatin Remodelers
Source: PLoS Genet. 2012 Oct 4;8(10):e1002994. doi: 10.1371/journal.pgen.1002994 (PMC3464200; doi:10.1371/journal.pgen.1002994)

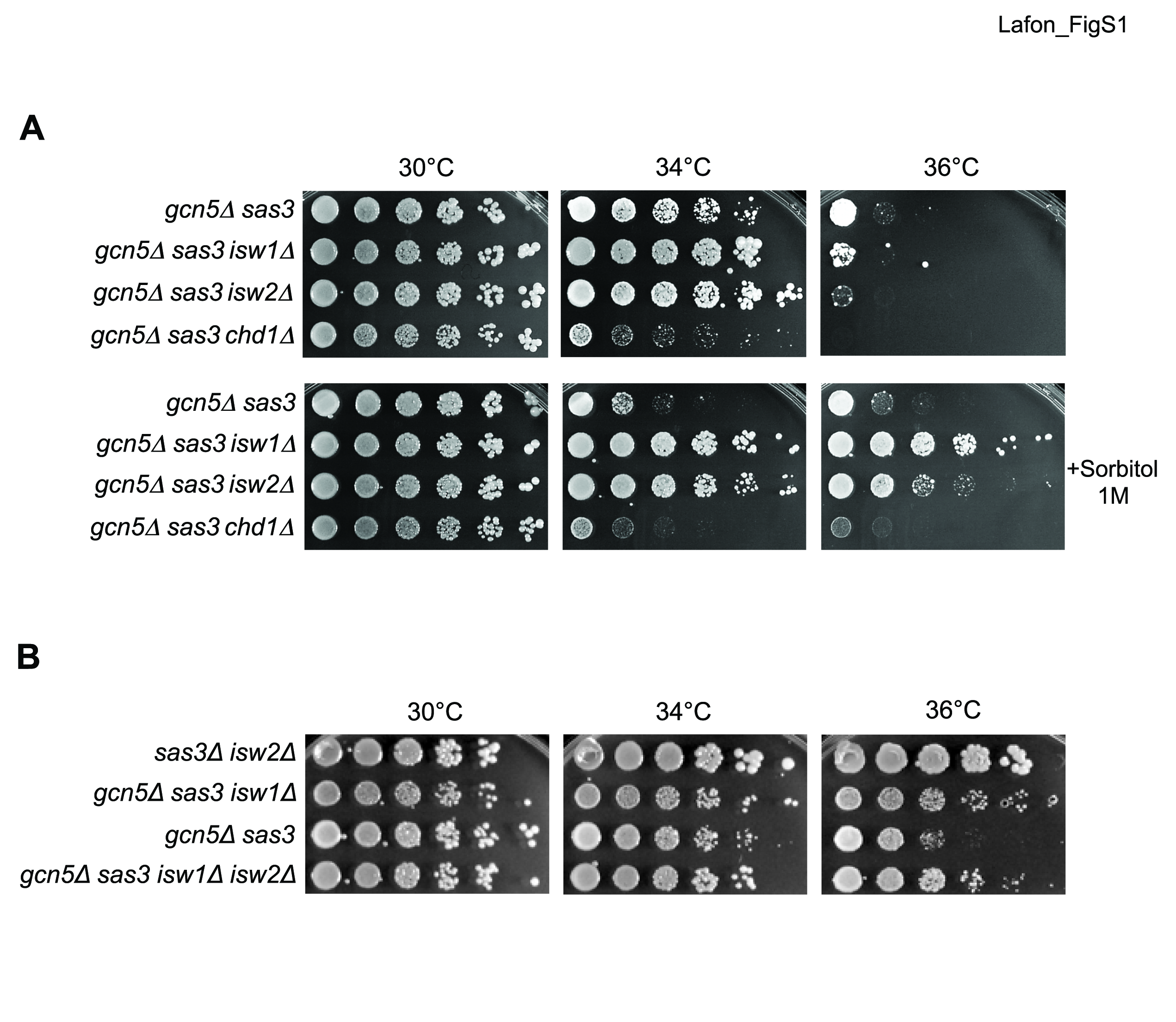

Supplement: Figure S1 — Functional interactions between Sas3 and Gcn5 acetyltransferases and chromatin remodeling enzymes ISWI and Chd1. (A) Inactivation of the Chd1 chromatin remodeling enzyme exacerbates the growth defects of the gcn5Δ sas3 mutant. Five-fold serial dilutions of cells were plated onto SC medium supplemented when indicated with 1 M sorbitol, and grown for 4 days at the indicated temperatures (see also Figure 1A). (B) Inactivation of the chromatin remodeling enzyme Isw2 does not further rescue the growth defects of the gcn5Δ sas3 isw1Δ mutant. Strains were plated onto SC medium supplemented with 1 M sorbitol, and grown at the indicated temperatures. (TIF) [file pgen.1002994.s001.tif]
