# Supplementary material for: Functional Antagonism between Sas3 and Gcn5 Acetyltransferases and ISWI Chromatin Remodelers
Source: PLoS Genet. 2012 Oct 4;8(10):e1002994. doi: 10.1371/journal.pgen.1002994 (PMC3464200; doi:10.1371/journal.pgen.1002994)
Supplement: Table S1 — Yeast strains used in this study. (DOC) [file pgen.1002994.s007.doc]

**Table S1. Yeast strains used in this study**

**Strain Genotype Reference**

**LPY5(W303-1a)** *MATa ade2-1 can1-100 his3-11 leu2,3,112 trp1-1 ura3-1* R. Rothstein

**LPY79** *MATα ade2-1 can1-100 his3-11 leu2,3,112 trp1-1 ura3-1*

**LPY13321** *MATα ade2-1 can1-100 his3-11 leu2-3,112 trp1 sas3∆::HIS3*

*gcn5∆::NatMX ura3-1::sas3 C357Y, P375A-URA3*

**LPY13140** *MATa ade2-1 can1-100 his3-11 leu2-3,112 trp1 sas3∆::HIS3 gcn5∆::KanMX ura3.1::sas3 C357Y, P375A -URA3* *isw1∆::kanMX*

**LPY14611** *MATα ade2-1 can1-100 his3-11 leu2-3,112 trp1 sas3∆::HIS3 gcn5∆::KanMX ura3.1::sas3C357Y, P375A -URA3 isw1∆::kanMX*

**LPY13816** *MATα ade2-1 can1-100 his3-11 leu2-3,112 trp1 gcn5∆::NatMX sas3∆::HIS3 ura3-1::sas3C357Y, P375A-URA3 isw2∆::KanMX*

**LPY13819** *MATα ade2-1 can1-100 his3-11 leu2-3,112 trp1 gcn5∆::NatMX sas3∆::HIS3 ura3-1::sas3C357Y, P375A-URA3 chd1∆::KanMX*

**LPY14836** *MATα ade2-1 can1-100 his3-11 leu2-3,112 trp1 gcn5∆::NatMX sas3∆::HIS3 ura3-1::sas3C357Y, P375A-URA3 isw1K227R*

**LPY15248**  *MATα ade2-1 can1-100 his3-11 leu2-3,112 trp1 gcn5∆::NatMX sas3∆::HIS3 ura3-1::sas3C357Y, P375A-URA3 isw2K215R-KanMX*

**LPY13028** *MATα ade2-1 can1-100 his3-11 leu2-3,112 trp1 sas3∆::HIS3 gcn5∆::KanMX ura3.1::sas3C357Y, P375A -URA3 ioc3∆::natMX*

**LPY15273** *MATα ade2-1 can1-100 his3-11 leu2-3,112 trp1 sas3∆::HIS3 gcn5∆::KanMX ura3.1::sas3C357Y, P375A -URA3 ioc2∆::kanMX*

**LPY13120** *MATα ade2-1 can1-100 his3-11 leu2-3,112 trp1 sas3∆::HIS3 gcn5∆::KanMX ura3.1::sas3C357Y, P375A -URA3 ioc4∆::hphMX*

**LPY13435** *MATα ade2-1 can1-100 his3-11 leu2-3,112 trp1 ura3-1 gcn5∆::natMX*

**LPY15247** *MATα ade2-1 can1-100 his3-11 leu2-3,112 trp1 gcn5∆::KanMX isw1∆::kanMX*

**LPY14566** *MATα ade2-1 can1-100 his3-11 leu2-3,112 trp1 gcn5∆::KanMX ioc3∆::natMX*

**LPY13634** *MATa ade2-1 can1-100 his3-11 leu2-3,112 trp1-1 ura3-1 IOC3-13Myc::KanMX*

**LPY13640** *MATa ade2-1 can1-100 his3-11 leu2-3,112 trp1 gcn5∆::NatMX IOC3-13Myc::KanMX*

**LPY13636** *MATa ade2-1 can1-100 his3-11 leu2-3,112 trp1 sas3∆::HIS3 gcn5∆::NatMX ura3-1::sas3C357Y, P375A-URA3 IOC3-13Myc::KanMX*

**LPY12932** *MATa ade2-1 can1-100 his3-11 leu2-3, 112 trp1-1 ^GAL sas3Δ::HIS3 isw2Δ::LEU2*

**LPY17152** *MATα ade2-1 can1-100 his3-11 leu2-3,112 trp1-1 sas3Δ::HIS3 gcn5Δ::NatMX ura3.1::sas3 C357Y, P375A-URA3 isw1Δ::KanMX isw2Δ::LEU2*

**LPY13395** LPY13321 + pLP1524 (*GCN5-LEU2* 2μ)

**LPY13396** LPY13321 + pLP645 (*SAS3-LEU2* 2μ)

**LPY13397** LPY13321 + pLP135 (*LEU2* 2μ)

**LPY13398** LPY13321 + pLP2234 (*IOC2-LEU2* 2μ)

**LPY13399** LPY13321 + pLP2260 (*IOC4-LEU2* 2μ)

**LPY13400** LPY13321 + pLP2266 (*IOC3-LEU2* 2μ)

**LPY13401** LPY13321 + pLP2256 (*ISW1-LEU2* 2μ)

**LPY15091** LPY13028 + pLP1524 (*GCN5-LEU2* 2μ)

**LPY15090** LPY13028 + pLP645 (*SAS3-LEU2* 2μ)

**LPY15089** LPY13028 + pLP135 (*LEU2* 2μ)

**LPY15092** LPY13028 + pLP2234 (*IOC2-LEU2* 2μ)

**LPY15094** LPY13028 + pLP2260 (*IOC4-LEU2* 2μ)

**LPY15275** LPY15273 + pLP1524 (*GCN5-LEU2* 2μ)

**LPY15276** LPY15273 + pLP645 (*SAS3-LEU2* 2μ)

**LPY15277** LPY15273 + pLP135 (*LEU2* 2μ)

**LPY15278** LPY15273 + pLP2266 (*IOC3-LEU2* 2μ)

**LPY15279** LPY15273 + pLP2256 (*ISW1-LEU2* 2μ)

**LPY13186** LPY13120 + pLP1524 (*GCN5-LEU2* 2μ)

**LPY13185** LPY13120 + pLP645 (*SAS3-LEU2* 2μ)

**LPY13184** LPY13120 + pLP135 (*LEU2* 2μ)

**LPY13190** LPY13120 + pLP2266 (*IOC3-LEU2* 2μ)

**LPY13189** LPY13120 + pLP2256 (*ISW1-LEU2* 2μ)

**LPY****13208** LPY13140 + pLP1524 (*GCN5-LEU2* 2μ)

**LPY13209** LPY13140 + pLP645 (*SAS3-LEU2* 2μ)

**LPY13207** LPY13140 + pLP135 (*LEU2-* 2μ)

**LPY13210** LPY13140 + pLP2234 (*IOC2-LEU2* 2μ)

**LPY13211** LPY13140 + pLP2260 (*IOC4-LEU2* 2μ)

**LPY13212** LPY13140 + pLP2266 (*IOC3-LEU2* 2μ)

**LPY13403** LPY13319 + pLP1524 (*GCN5-LEU2* 2μ)

**LPY13404** LPY13319 + pLP645 (*SAS3-LEU2* 2μ)

**LPY13405** LPY13319 + pL135 (*LEU2-* 2μ)

**LPY13406** LPY13319 + pLP2234 (*IOC2-LEU2* 2μ)

**LPY17343** LPY13634 + pLP 135 (*LEU2* 2μ)
**LPY17344** LPY13634 + pLP 2234 (*IOC2-LEU2* 2μ)
**LPY17345** LPY13634 + pLP 2260 (*IOC4-LEU2* 2μ)

**LPY17346**  LPY13636 + pLP 135 (*LEU2* 2μ)
**LPY17347** LPY13636 + pLP 2234 (*IOC2-LEU2* 2μ)
**LPY17348** LPY13636 + pLP 2260 (*IOC4-LEU2* 2μ)
