# Supplementary material for: Functional Antagonism between Sas3 and Gcn5 Acetyltransferases and ISWI Chromatin Remodelers
Source: PLoS Genet. 2012 Oct 4;8(10):e1002994. doi: 10.1371/journal.pgen.1002994 (PMC3464200; doi:10.1371/journal.pgen.1002994)
Supplement: Table S2 — ChIP primers used in this study. (DOCX) [file pgen.1002994.s008.docx]

**Table S2. ChIP primers used in this study**

**Region**  **Primer** **Sequence Source**

***PYK1* promoter** OLP1132 5’-TCCTTTCCTTCCCATATGAT-3’

-298-123 OLP1133 5’-AACTTTGAAAGGGGACCATG-3’

***PYK1* promoter** OLP1336 5’-TCCTTTCCTTCCCATATGATGC-3’

-298-218 OLP1343 5’-GAATATCGTTTTGATGGCGAGC-3’

***PYK1* 5’ region** OLP1105 5’-TGGTTGCTTTGAGAAAGGCTGG-3’

+110+324 OLP1098 5’-TTCGTGGTTTGGTGGGATTGG3-’

***PYK1* 3’ region** OLP1121 5’-TGGTTACCAGATGCCCAAGAG-3’

+1265+1458 OLP1122 5’-CTTGAAACCTTGGATGGAAACG-3’

***PMA1* 5’ region** OLP1188 5’-TCAGCTCATCAGCCAACTCAAG -3’ (1)

+32+123 OLP1189 5’-CGTCGACACCGTGATTAGATTG-3’ (1)

***RPL10* promoter** OLP1151 5’-TCCCTCCGAAACTAGTTAGCACAA-3’

-200+18 OLP1152 5’-AGCTGGTCTTCTAGCCATCTTGAA-3’

***RPL10* 5’ region** OLP1214 5’-ACCAAAAGAACAAGCCTTACCC-3’

+32+123 OLP1215 5’-AGCCTTCTTCTTACCCAAATCG-3’

***RPS5* 5’ region** OLP1210 5’-CTGACACCGAAGCTCCAGTTGAAG-3’

+5+103 OLP1211 5’-GTTGGACTTCTTCTGGAATTGGAG-3’

***UBP7* promoter** OLP1156 5’-ACTTGTGGCTCCTCGACGTATTTC-3’

-152-24 OLP1157 5’-GCGATAGCTCTTTAAGTTGCGG-3’

***UBP7* 5’ region** OLP1218 5’-TGCTAGACGATGATAAGGGCAC-3’

+2+91 OLP1219 5’-CCTGGACCCTTCGCAGTAATTC-3’

***CDC25* promoter** OLP1176 5’-ATAGTCGACGTCAGTCACCTCCAG-3’

-324-161 OLP1177 5’-CCGTGTTGGTGATGCAGTTTTC-3’

***CDC25* 5’ region** OLP1216 5’-AGGCAGGCAATGCTTCACAAAC-3’

+47+146 OLP1217 5’-GGGAAGAAGAAAGAGAAGCTGG-3’

**rDNA 5S** OLP776 5’-CATGGAGCAGTTTTTTCCGC-3’

OLP777 5’-TACAAGCACTCATGTTTGCCG-3’

**TelVIR** OLP778 5’-AAATGGCAAGGGTAAAAACCAG-3’

OLP779 5’-TCGGATCACTACACACGGAAAT-3’

***ACT1*** OLP798 5’-GGTGGTTCTATCTTGGCTTC-3’

+1024-1102 OLP799 5’-ATGGACCACTTTCGTCGTAT-3’

***PYK1* 1** OLP1709 5’-CAGATTGGGAGATTTTCATAGTAGA-3’

-499-379 OLP1710 5’-CACTCAACTCAACTGAACG-3’

***PYK1* 2** OLP1711 5’-ATGTGTTCCGCACCGTCA-3’

-452-352 OLP1712 5’-TTTAGCTAAGATCCATTGAACAAA-3’

***PYK1* 3** OLP1713 5’-TTCGTTCATTCAGTTGAGTTGAG-3’

-406-305 OLP1714 5’-TTGGAAGACATCACAAGCATTCT-3’

***PYK1* 4** OLP1715 5’-TTTTTCTCTTGGTAAATGAATGCTT-3’

-344-254 OLP1716 5’-TAGGAAGACACTAAAGGTACCTAGCA-3’

***PYK1* 5** OLP1717 5’-TCCTTTCCTTCCCATATGAT-3’

-299-209 OLP1718 5’-AAAAGCCAACGAATATCGTTTGA-3’

***PYK1* 6** OLP1719 5’-AAAGGCTCGCCATCAAAAC-3’

-245-155 OLP1720 5’-AAAAGAGGTTCTTGGAAATGAAAA-3’

***PYK1* 7** OLP1721 5’-AAAGGCTCGCCATCAAAAC-3’

-191-86 OLP1722 5’-AAAAGAGGTTCTTGGAAATGAAAA-3’

***PYK1* 8** OLP1727 5’-ACAAGACACCAATCAAAACAAA-3’

-37-+53 OLP1728 5’-AAGTCAGAACCAGCAACAACG-3’

***PYK1* 9** OLP1729 5’-TGACCTCATTAAACGTTGTTGC-3’

+20-109 OLP1730 5’-AGGTTTCTGGGTTGTTGGTC-3’

***PYK1* 10** OLP1731 5’-GGTACCATCGGTCCAAAGAC-3’

+72-167 OLP1732 5’- GAACCGTGAGAGAAGTTCATACG-3’

***PYK1* 11** OLP1735 5’-CACGGTTCTTACGAATACCACA-3’

+159-262 OLP1736 5’-GACCCTTGGTGTCCAAAGC-3’

***PYK1* 12** OLP1737 5’-GAAAGTCCGAAGAATTGTACCC-3’

+202-298 OLP1738 5’-CAACATCGTTGGTGGTGGTA-3’

***PYK1* 13** OLP1739 5’-CCAAGGGTCCAGAAATCAGA-3’

+253-352 OLP1740 5’-CGTACTTGTCATCGGTGGTG-3’

***PYK1* 14** OLP1741 5’-GACTACCCAATCCCACCAAA-3’

+297-403 OLP1742 5’- TGGTGATGTTCTTGTAGTCAACG-3’

***PYK1* 15** OLP1743 5’- CCGATGACAAGTACGCTAAGG-3’

+337-427 OLP1744 5’- TGATTCTACCAGCGGAGATG-3’

***PYK1* 16** OLP1745 5’**-** TGACTACAAGAACATCACCAAGG-3’

+383-483 OLP1746 5’- CTTGTCGTCAACGACTTCCA-3’

***PYK1* 17** OLP1747 5’**-** ACGTTGATGATGGTGTTTTGTC-3’

+430-520 OLP1748 5’- AGATCTTACCGGCGTTCAAA-3’

***PHO5* TATA** OLP1780 5’-CCATTTGGGATAAGGGTAAACA-3’

-147-68 OLP1781 5’-AACCTCGACTTAGCAAAACATC-3’

1. Vitaliano-Prunier A*, et al.* (2008) Ubiquitylation of the COMPASS component Swd2 links H2B ubiquitylation to H3K4 trimethylation. *Nat Cell Biol* 11:1365-1371.
