# Supplementary material for: Functional Antagonism between Sas3 and Gcn5 Acetyltransferases and ISWI Chromatin Remodelers
Source: PLoS Genet. 2012 Oct 4;8(10):e1002994. doi: 10.1371/journal.pgen.1002994 (PMC3464200; doi:10.1371/journal.pgen.1002994)
Supplement: Text S1 — Supporting Methods. (DOCX) [file pgen.1002994.s009.docx]

**SI Material and Methods**

Protein Immunoblotting

Whole cell extracts were prepared as previously described [1]. Briefly, cells were grown in minimal medium at the indicated temperature to an A_600_ of 0.7-0.9. Cells were lysed with glass beads in phosphate buffered saline with protease inhibitors, resuspended in loading buffer, and separated by 18% (for histones) or 7% (for Ioc3-myc) sodium dodecyl sulfate-polyacrylamide gel electrophoresis (SDS-PAGE). Proteins were transferred to nitrocellulose membranes, blotted with either anti-H3 (07-690, Upstate/Millipore) and anti-acetylated H3K14 (07-353, Upstate/Millipore) or anti-myc (9E10) and anti-tubulin [2]. Blots were then incubated with horseradish peroxidase-conjugated anti-rabbit (1:10,000, Promega) or anti-mouse (1:10,000, Promega) and developed by enhanced chemiluminescence (Perkin-Elmer). Image J software (<http://rsbweb.nih.gov/ij/>) was used for quantification using a detailed protocol outlined by Luke Miller (Stanford University) (<http://lukemiller.org/index.php/2010/11/analyzing-gels-and-western-blots-with-image-j/>).

mRNA quantification

50 ml cultures of wild type, *gcn5Δ sas3*, and *gcn5Δ sas3 ioc3Δ*, were grown in SC at 34°C to OD 0.8-1.0. Cells were collected by centrifugation, resuspended in 450 μl AE buffer, pH 5.0 (50 mM sodium acetate, pH 5.3, 10 mM EDTA, pH 8.0 in nuclease free water, pH adjusted with acetic acid) and 50 μl 10% SDS and vortexed for 15 seconds. 500 μl of phenol equilibrated with AE buffer was added and cells were vortexed again for 15 seconds followed by incubation at 65°C for four minutes. Samples were incubated on ice for 60 seconds followed by centrifugation at top speed for three minutes. The aqueous layer was removed followed by extraction with AE-equilibrated phenol and chloroform. Nucleic acids were precipitated overnight and resuspended in 100 μl nuclease free water. RNA quality was determined by agarose gel electrophoresis and analysis at A_260_ and A_280_. The TURBO DNA-free kit by Ambion was used to digest DNA. 1 μg of RNA was used for cDNA synthesis using the TaqMan RT Kit from ABI (including reactions minus reverse transcriptase).

The cDNA was freshly diluted to 1:10 for use as template in qPCR reactions to measure *ACT1*, *PYK1*, *PMA1*, and *RPL10* expression. Primers used are listed in Table 2. *PYK1*, *PMA1*, and *RPL10* levels were determined, relative to *ACT1* and the WT ratio was set to 1.

Chromatin analysis

Extracts from MNase digestions were prepared as described [3,4]. Briefly, cultures were grown in SC medium at 34^o^C to an A_600_ of 0.7-0.9. Then ~ 2x10^9^ cells were harvested, washed in 1 ml sorbitol 1M, resuspended in 1 ml of zymolyase solution (sorbitol 1.1M, 20 mM KPO_4_, pH7, 0.5 mM CaCl_2_, β-mercaptoethanol 0.5 mM, zymolyase 100T 1 mg/ml) and incubated for 1.5 min at room temperature. Spheroplasts were then washed twice in 1M sorbitol and gently resuspended in 1.6 ml of cold buffer A (1 M sorbitol, 50 mM NaCl, 10 mM Tris-HCl, pH 7.4, 5 mM MgCl_2_, 0.5 mM spermidine, 0.075% NP40 and 1 mM β-mercaptoethanol). The cell slurry was divided into 400 μl aliquots, each one added to a microfuge tube containing the MNase (0, 60, 150 and 400 U/ml final concentrations) and incubated at 37^o^C for 4 minutes. The reaction was stopped by addition of 40 μl of stop buffer (250 mM EDTA, 5% SDS). DNA purification was performed as described in [4]. The control naked DNA was digested with 30 U/ml MNase for 1 minute at 37^o^C. Samples of purified genomic DNA were digested with various restriction enzymes (EcoRI, BglII, XbaI, StyI) and pooled to provide molecular weight markers. MNase extracts were then digested to completion with EcoRI and analyzed by indirect end labeling. The probe for *PYK1* locus was PCR amplified from genomic DNA using OLP1358 and OLP1359, digested with the restriction enzymes EcoRI and StyI and gel purified before [α-^32^P] dCTP radiolabeling by random priming (Random Primers DNA Labeling System, Invitrogen).

1. Clarke AS, Lowell JE, Jacobson SJ, Pillus L (1999) Esa1p is an essential histone acetyltransferase required for cell cycle progression. Mol Cell Biol 19: 2515-2526.

2. Bond JF, Fridovich-Keil JL, Pillus L, Mulligan RC, Solomon F (1986) A chicken-yeast chimeric beta-tubulin protein is incorporated into mouse microtubules in vivo. Cell 44: 461-468.

3. Kent NA, Mellor J (1995) Chromatin structure snap-shots: rapid nuclease digestion of chromatin in yeast. Nucleic Acids Res 23: 3786-3787.

4. Wu L, Winston F (1997) Evidence that Snf-Swi controls chromatin structure over both the TATA and UAS regions of the *SUC2* promoter in *Saccharomyces cerevisiae*. Nucleic Acids Res 25: 4230-4234.
